# Erdheim-Chester Disease in Adults: 11 Cases from a Canadian Rare Diseases Program

**DOI:** 10.46989/001c.145180

**Published:** 2025-10-18

**Authors:** Stephanie Quon, Emily Leung, Mark Trinder, Liliana Wolak, Mariam Goubran, Fatimah Al-Ani, Maude Landry, Mollie Carruthers, Luke Y.C. Chen

**Affiliations:** 1 Vancouver Fraser Medical Program, Faculty of Medicine, University of British Columbia, Vancouver, BC, Canada; 2 Department of Medicine, University of British Columbia, Vancouver, BC, Canada; 3 Department of Pathology and Laboratory Medicine, University of British Columbia, Vancouver, BC, Canada; 4 Department of Radiology, University of British Columbia, Vancouver, BC, Canada; 5 Division of Hematology, University of British Columbia, Vancouver, BC, Canada; 6 Coastal Rare Inflammatory Diseases Program, BC/NS, Canada; 7 Horizon Health Network, Moncton, NB, Canada; 8 Division of Hematology, Dalhousie University, Halifax, NS, Canada; 9 Division of Hematology, Université de Sherbrooke, Sherbrooke, QC, Canada; 10 Arthritis Research Center, Vancouver, BC, Canada

**Keywords:** histiocytosis, rare diseases, clonal hematopoiesis, targeted therapy, systemic inflammation, FDG-PET imaging

## Abstract

Erdheim-Chester disease (ECD) is a rare histiocytic neoplasm with highly variable, multisystem manifestations that present significant diagnostic and therapeutic challenges. This retrospective multicenter case series included 11 adult patients diagnosed with biopsy-proven ECD across Canada between January 2015 and June 2024. The cohort comprised six females and five males with a median age of 55 years (range 41-74). PET-CT was used for disease staging and treatment monitoring in nine cases. The most commonly involved sites were bone (n=8), kidney (n=6), and lungs (n=5). BRAF V600E mutations were detected in seven patients. Treatments included vemurafenib, interferon, tocilizumab, cladribine, cobimetinib, and cytarabine. Treatment responses varied, with several patients achieving remission or stable disease, while others had progressive or end-stage disease. This study highlights the clinical heterogeneity of ECD and the value of integrating histopathology, molecular profiling, and imaging to guide management and improve outcomes.

## Introduction

Erdheim-Chester disease (ECD) is a rare, “L group” histiocyte disorder first described in 1930 by Jakob Erdheim and William Chester.^1,2^ The disease is characterized by xanthogranulomatous infiltration of tissues by foamy, lipid-laden histiocytes that are CD68+ and CD1a-.^1,2^ Since its discovery, ECD has remained an under-recognized condition due to its rarity and highly variable presentation.^3,4^ Incidence is estimated at one case per two million per year^5^ with a median age of diagnosis in the fifth to seventh decades of life and a slight male predominance.^3,6^

Clinically, ECD most frequently affects the long bones, leading to bilateral, symmetric osteosclerosis in over 90% of cases.^7–9^ Other hallmark features include diabetes insipidus, retroperitoneal fibrosis, and perinephric soft tissue infiltration.^2,8,10^ Cardiovascular involvement is seen in up to 50% of cases, and central nervous system (CNS) infiltration, particularly meningeal and vascular lesions, is associated with adverse outcomes.^8,11^ While this constellation of clinical features is highly specific to ECD, limited awareness of the disease often leads to delays in diagnosis and management.^3,11^

Diagnosing ECD requires a comprehensive, multidisciplinary approach that integrates clinical, radiologic, histopathologic, and molecular findings.^8,12^ Histologically, the disease is characterized by foamy histiocytes and Touton giant cells within a fibrotic and inflammatory background. These features can be subtle and are often missed on core needle biopsies. As a result, multiple biopsies are frequently required to establish a definitive diagnosis.^2,12^ Radiologically, imaging modalities such as 18F-fluorodeoxyglucose positron emission tomography (FDG-PET/CT) are valuable, as they can detect metabolically active lesions that may not be apparent on standard CT scans. This can aid in identifying optimal sites for biopsy.^13,14^

This study presents our experience with ECD in a Canadian rare disease program. Much of the literature on this rare disease originates from centers of excellence specializing in histiocytic disorders, where advanced diagnostic and therapeutic resources are readily available. As we have previously done with Rosai-Dorfman-Destombes disease,^15,16^ we sought to characterize the clinical features, diagnostic challenges, and treatment outcomes of patients within a Canadian “real-world” setting, where such specialized resources may be limited.

## Methods

This was a retrospective study of 11 consecutive adult patients (age > 17 years) with a diagnosis of ECD between January 2015 and June 2024 managed at the Coastal Rare Inflammatory Diseases Program. This program is directed by the senior author, and provides diagnostic and treatment advice for patients with rare diseases across Canada, who are then treated in their own center. Patient information is housed at Vancouver General Hospital, and ethics approval was obtained from the University of British Columbia Clinical Research Ethics Board (H23-01853). The majority of patients (n=7) were based in British Columbia, Canada, while the remaining were from Nova Scotia (n=2), New Brunswick (n=1) and Quebec (n=1). One of the patient cases (case 6, as described in Tables 2 and 3) was previously described in an educational case report (24).

Data were extracted from electronic medical records and included demographics (age, gender), clinical presentation (symptoms, organ systems involved), histopathology, imaging results, treatments administered and their response, follow-up duration, and survival status. The data cut-off date was June 30, 2025. Definitive histopathological diagnoses were confirmed by tissue biopsy and reviewed by pathologists from BC Cancer. Follow-up duration was calculated from the date of disease diagnosis to the date of the last follow-up by the primary clinician managing the patient’s ECD care. Treatment response was evaluated through clinical assessment and radiographic imaging.

## Results

### Patient Characteristics, Presenting Features, and Organ Involvement

This retrospective case series included 11 adult patients with a histopathological diagnosis of ECD. Demographics and time to diagnosis are shown in Table 1.

**Table 1.** Summary of patient and disease features.

*Feature*	*Value*
Total patients	11
Median age at diagnosis (years)	55 (range 41-74)
Gender (female:male)	6:5
Median months from symptom onset to diagnosis	12 (range 3-144)
Median number of biopsies for diagnosis	2 (range 1-5)
Median years of follow-up	4.0 (range 1.6-9.2)
Deaths	1
*Organ involvement*	
Bone	n=8 (73%)
Kidneys	n=6 (55%)
Lungs	n=5 (46%)
Heart	n=5 (46%)
CNS	n=5 (46%)
Lymph nodes	n=3 (27%)
Retroperitoneum	n=2 (18%)
Eye	n=2 (18%)
Skin	n=2 (18%)
Adrenal gland, liver, sinuses, pancreas, spleen, gonads	n=1 each (10%)

Presenting symptoms were most commonly pain or swelling in the lower extremities, back, or flank (n=6), followed by neurologic manifestations such as dizziness, dysarthria, ataxia, numbness or tingling, syncopal episodes, and decreased level of consciousness (n=5). Endocrine-related symptoms included central diabetes insipidus and polydipsia (n=4). Dermatologic findings such as xanthelasmas and yellow discoloration of the skin were reported in a subset of patients (n=2). Other reported features included lymphadenopathy (n=1), anemia (n=1), transfusion-dependent cytopenias (n=1), pericardial effusion (n=1), decreased muscle mass (n=1), low libido (n=1), jaw and oral symptoms (n=1), and urinary obstruction (n=1). None of the patients with cytopenias had evidence of an associated myeloid neoplasm.

Rates of organ involvement determined by imaging suggestive of infiltration are shown in Table 1. Organ involvement was most commonly bone (n=8), kidney (n=6), and lungs (n=5). Lymph node involvement was seen in four patients. A detailed breakdown of presenting symptoms and organ involvement per patient is outlined in Table 2.

**Table 2.** Individual patient characteristics, presenting features of ECD, and organ involvement.

*Case*	*Age at Diagnosis*	*Gender*	*Presenting Symptoms*	*Organ Involvement*	*Lymph Node Involvement*
1	54	Male	Increasing back and flank pain, urinary obstruction	Bones, heart, kidneys, lungs, CNS, ureters, retroperitoneum	No
2	74	Female	Episodes of decreased level of consciousness, transfusion-dependent cytopenias	Bone marrow	No
3	52	Female	Pain and swelling in the lower extremities	Bones, eyes, heart, kidneys, lymph nodes, lungs, spleen, ureters	Yes
4	66	Male	Polydipsia, numbness and tingling in the legs	Heart, kidneys, lungs, testicle	No
5	41	Male	Lower extremity bone pain bilaterally, syncopal episodes, dysarthria, ataxia, xanthelasmas, decreased muscle mass, low libido	Bones, eye, gonads, kidneys, lungs, pituitary, skin	No
6	52	Female	Dysarthria, yellow discoloration of skin on temples, inner eyes, and forehead, ataxia, lymphadenopathy	CNS, lungs, skin	No
7	61	Female	Pain and swelling in lower extremities, difficulty walking with heaviness in legs	CNS, heart, kidneys, retroperitoneum	No
8	44	Male	Lower extremity swelling, neck stiffness	Bone, lymph nodes, adrenal gland, bone marrow	Yes
9	65	Female	Central diabetes insipidus, dizziness, edema	CNS, liver, bone, bone marrow, lungs, lymph nodes	Yes
10	67	Female	Anemia, pericardial effusion, bone pain	Bone, heart, sinuses, kidneys	No
11	55	Male	Upper palate burning, tooth loss, gum atrophy, weight loss, mixed LCH/ECD	Bone, palate, CNS, pancreas	No

Recent molecular cluster analyses have stratified ECD into three clinical-molecular subtypes: the Widespread Disease (WID) cluster, associated with BRAFV600E mutations and extensive multi-organ involvement; the Limited Disease (LIM) cluster, typically BRAFV600E-negative or anatomically restricted; and the MAP2K1-RDD (MAP) cluster, overlapping with MAP2K1 pathway mutations and Rosai-Dorfman-Destombes disease.^11^ In our cohort, nine patients (82%) were classified as WID and two (18%) as LIM.

### Histopathologic Features, Imaging, and Laboratory Findings

Histopathological confirmation of ECD was obtained through a median of two biopsies (range 1-5). The sites biopsied as part of the diagnostic workup included the retroperitoneal or perirenal region (n=5), followed by bone marrow (n=3) and long bones such as the femur or tibia (n=3). Additional sites included the skin (n=2), lymph nodes (n=2), and soft tissue or muscle (n=2). Less frequently biopsied sites were the duodenum (n=1), liver (n=1), and oral mucosa (n=1).

Histologic findings were most commonly consistent with histiocytic infiltration (n=10) and fibrosis (n=9), aligning with typical ECD pathology. Foamy macrophages were noted in four cases, often within fibroadipose or bone marrow tissue. A mixed inflammatory infiltrate, often comprising lymphocytes, plasma cells, and occasionally neutrophils, was observed in five cases, and markedly increased cellularity was present in two bone marrow samples.

**Figure 1.**
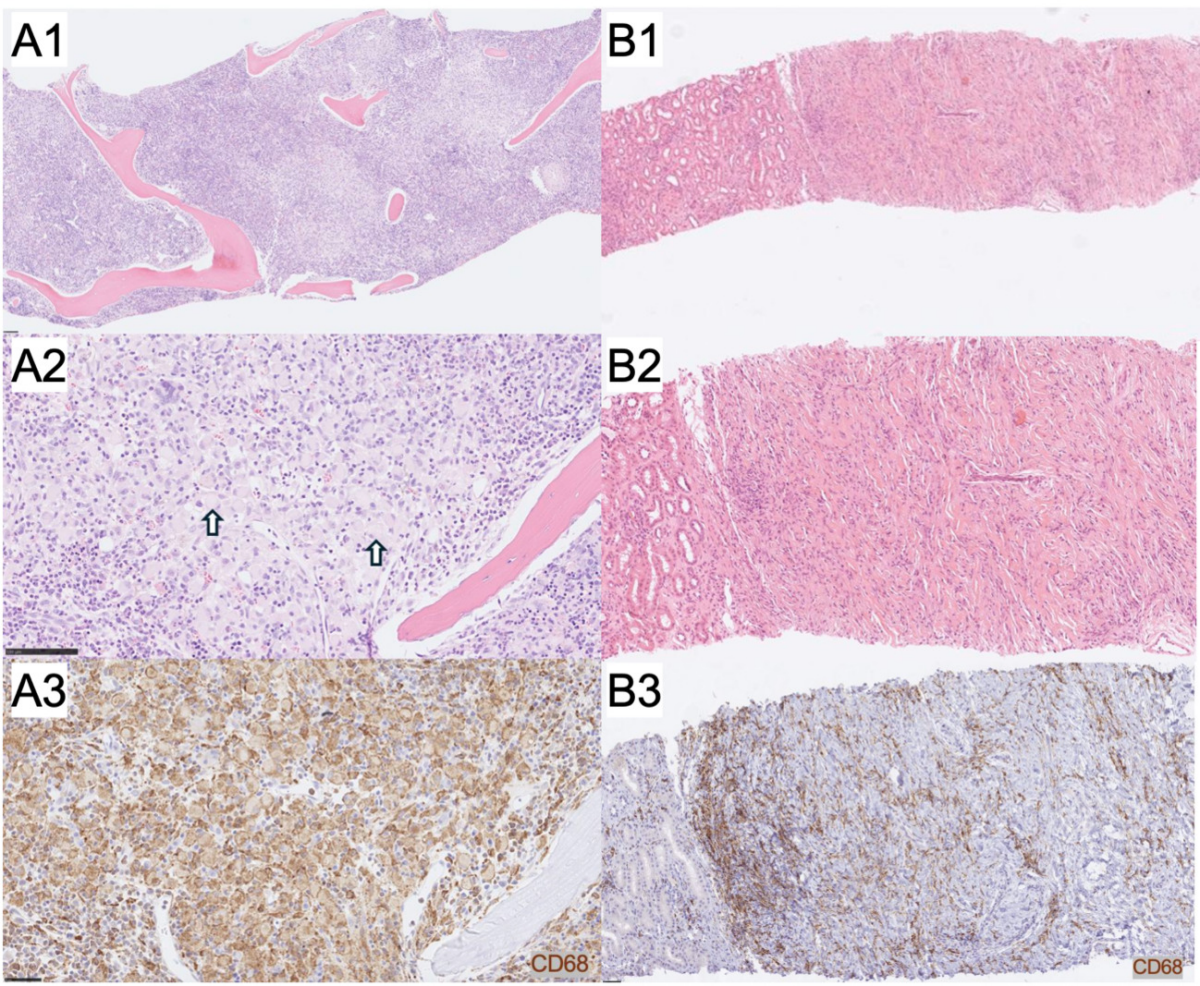
Histopathological and immunohistochemical features of ECD from two biopsy samples. The scale bars depict: (A1) 100 , (A2) 100, (A3) 50, (B1) 100 , (B2) 50, and (B3) 50 micrometers, respectively. A1-A2: Hematoxylin and eosin-stained sections of a bone marrow core biopsy of show classic findings of hypercellular proliferation and atypical histiocytes arranged in sheets between the intertrabecular space. The histiocytes display crescentic nuclei and single large cytoplasmic macrovesicles with focal xanthomatous appearance (white arrows) [Case 2]. A3: Immunohistochemistry shows these cells are positive for CD68, CD163, CD4, cyclin D1, factor XIIIA [Case 2]. B1-B2: Hematoxylin and eosin-stained sections of a perinephric needle biopsy show a moderately cellular, sclerosing fibroinflammatory lesion with a vague histiocytic appearance adjacent to uninvolved renal cortical parenchyma. The histiocytic cells do not display plentiful foamy/xanthomatous cytoplasm and no Touton giant cells are seen [Case 4]. B3: Immunohistochemistry shows the cells of the fibroinflammatory lesion are positive for CD68, CD163, and factor XIIIa and negative for for S100, CD21, CD23, and CD1a [Case 4].

Classic histopathologic features of ECD are demonstrated in a bone marrow core biopsy, showing sheets of atypical histiocytes with crescentic nuclei and focal xanthomatous cytoplasm occupying the intertrabecular space (**Figures 1A1 and 1A2**). In contrast, a perinephric needle biopsy illustrates a non-classical presentation, with subtle histiocytic infiltration embedded within a sclerosing fibroinflammatory background lacking prominent foamy cytoplasm (**Figures 1B1 and 1B2**).

Immunohistochemistry supported a histiocytic lineage in all tested cases. CD68 was positive in all interpretable cases, with staining ranging from strong and diffuse to patchy or scattered. Factor XIIIa was positive in four of five cases where it was tested, further supporting histiocytic involvement. In the single case with negative Factor XIIIa staining, the diagnosis of ECD was supported by characteristic clinical features, radiologic findings, and compatible immunohistochemical and molecular results (CD68+, CD1a-, S100-, and BRAF V600E+). CD1a is typically negative in non-Langerhans cell histiocytosis, serving as a distinguishing immunophenotypic feature. In our cohort, CD1a was negative in nine of ten tested cases. The single case with weak focal CD1a positivity involved palatal tissue from a patient who was concurrently diagnosed with Langerhans cell histiocytosis, suggesting possible tissue involvement by both entities or sampling from an area affected by LCH. S100 staining was negative in five of ten tested cases, and weak or minimal staining was noted in the five positive cases. BRAF V600E immunostaining was positive in seven patients.

Representative immunohistochemical staining patterns are shown in the bone marrow (**Figure 1A3**) and perinephric (**Figure 1B3**) biopsies. Both demonstrated strong CD68 and CD163 positivity, with CD163 tested in two patients overall and positive in both. The bone marrow biopsy additionally showed expression of factor XIIIa, CD4, and cyclin D1, while the perinephric biopsy lacked S100, CD1a, and follicular dendritic cell markers. These findings support a diagnosis of ECD over other histiocytic or fibrosing conditions such as Langerhans cell histiocytosis or IgG4-related disease.

Molecular analysis using next-generation sequencing was performed in 6 patients. BRAF V600E mutations were identified in four cases, with an additional patient showing BRAF K601E and BRAF F595L variants at low allele frequency. One patient demonstrated co-mutations in KRAS (D33E) and NRAS (G60E), and another harbored a CSF3R mutation without concurrent MAPK pathway alterations. In one case, BRAF immunohistochemistry was positive on tissue biopsy but no corresponding mutation was detected in blood.

Inflammatory markers were frequently elevated at the time of diagnosis, with a median C-reactive protein of 33.5 mg/L (range 9.5-178). Polyclonal hypergammaglobulinemia was observed in four patients. An overview of the histology and laboratory findings of each case is provided in Table 3.

**Table 3.** Next-generation sequencing and immunohistochemistry results of each case.^*^

	Next Generation Sequencing	Immunohistochemistry				
*Case*	*Mutations Detected (VAF%)*	*CD68*	*Factor XIIIa*	*BRAF V600E*	*S100*	*CD1a*
1	ND (NGS attempted, issue with sample)	+	ND	+	Focal weak +	-
2	BRAF V600E (6.7%), KRAS D33E (17.7%), NRAS G60E (3.4%)	+	+	+	Minor subset +	-
3	ND	+ (strong, foamy)	ND	+ (weak)	-	-
4	ND	+ (strong)	+ (strong)	-	-	-
5	ND	+ (bone)	-	+	-	-
6	BRAF V600E (VAF ND)	+	ND	+	focal weak +	-
7	ND	+ (patchy)	+	-	-	-
8	BRAF K601E (0.6%), BRAF F595L (0.6%) in bone marrow	+ (scattered)	+ (few cells)	-	-	-
9	BRAF V600E	+	+	+	Weak +	-
10	CSF3R mutation only; Oncomine panel otherwise negative	+ (mild)	ND	-	ND	ND
11	BRAF IHC positive in palate, V600E negative in blood	+	ND	+	+ (weak)	+ (weak)

* ND = no data; VAF = variant allele frequency; + = positive; - = negative.

**Figure 2.**
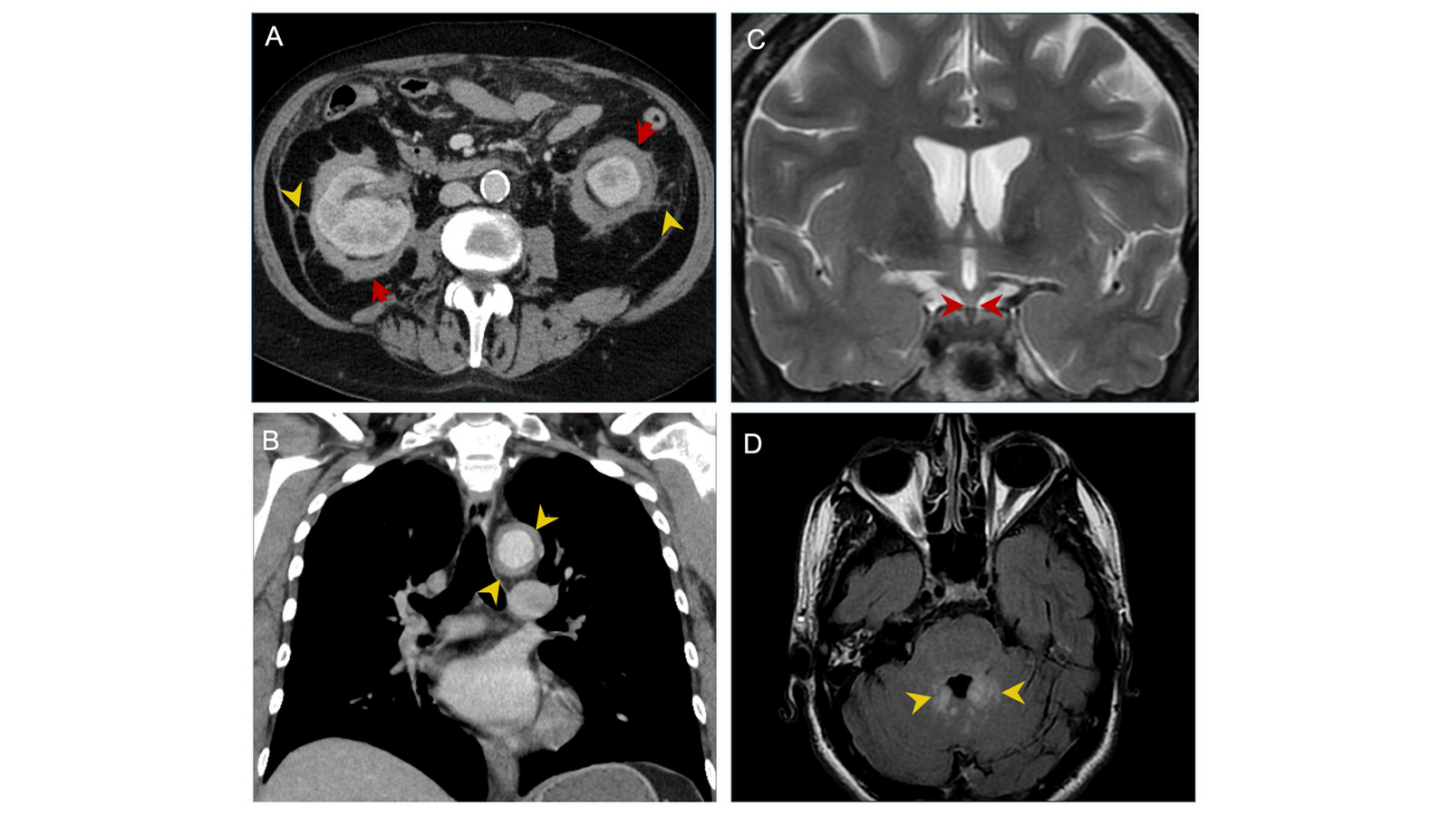
The retroperitoneal (A), cardiovascular (B), and neurologic (C, D) manifestations of ECD. A: Axial portal venous phase CT demonstrating soft tissue infiltration (yellow arrows) and perirenal septal thickening (arrowheads) known as the pathognomonic ‘hairy kidney sign’ [Case 4]. B: Coronal contrast-enhanced CT demonstrating the characteristic circumferential infiltration of the aorta (arrowheads) known as the ‘coated aorta sign’ [Case 3]. C: Coronal T2-weighted MRI demonstrating a thickened pituitary infundibulum (arrowheads) [Case 5]. D: Axial T2 FLAIR hyperintensities at the bilateral dentate nuclei within the cerebellum (arrowheads) [Case 5].

Of the 11 patients, nine were subjected to FDG PET-CT imaging. The most frequent finding was skeletal involvement (n=7), typically as sclerotic lesions or diffuse long bone infiltration. Renal and perinephric involvement was also common (n=6), including soft tissue infiltration and perirenal septal thickening consistent with the ‘hairy kidney sign’ (**Figure 2A**) and retroperitoneal fibrosis. Pericardial abnormalities, including effusion, thickening, or mass, were noted in three patients. One patient showed the classic ‘coated aorta sign,’ characterized by circumferential aortic infiltration (**Figure 2B**). Neurological findings were also observed, including a thickened pituitary infundibulum (**Figure 2C**) and bilateral cerebellar dentate hyperintensities (**Figure 2D**). Other frequently involved areas included the lymph nodes (n=3) and lungs (n=3). Less common FDG PET-CT findings included adrenal gland involvement (n=1), testicular FDG uptake (n=1), and gastric pylorus activity (n=1).

### Treatments and Outcomes

The median number of systemic therapies received per patient was 1 (range: 0-3). First-line treatments included vemurafenib (n=3), pegylated interferon alpha-2a (n=2), tocilizumab (n=2), cladribine (n=1), cobimetinib (n=1), and cytarabine (n=1). Second-line therapies included vemurafenib (n=2), tocilizumab (n=1), and cladribine (n=1). One patient also received pegylated interferon alpha-2a as a third-line agent. Three patients had not yet started systemic therapy by the time of data cut-off, as they had been recently diagnosed.

**Figure 3.**
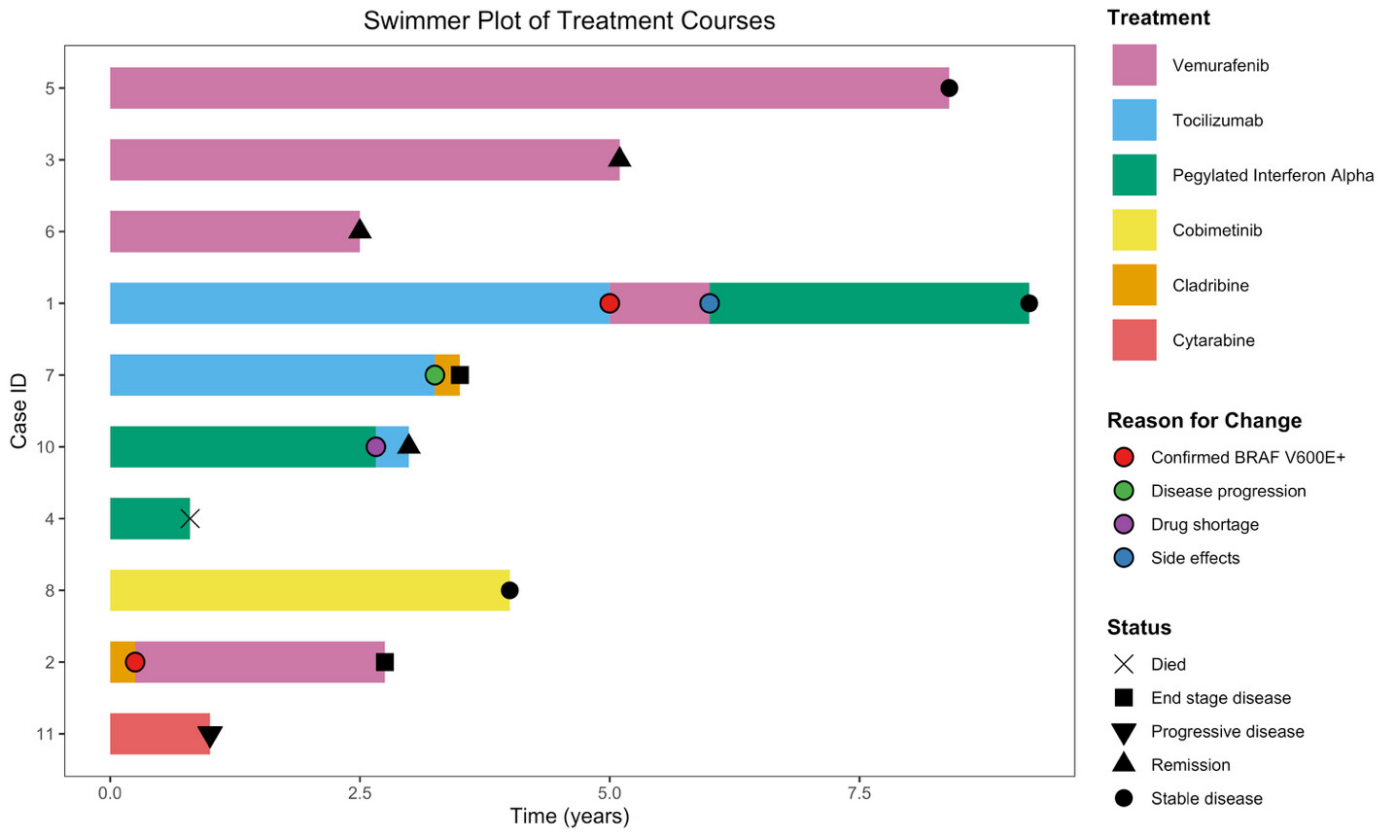
Swimmer plot of sequential treatments and outcomes in 10 patients with ECD.

The median follow-up duration was 3.9 years (range: 0.5-9.2 years). At last follow-up, of the 10 patients undergoing treatment, four had stable disease, three had progressive or end-stage disease, two were in remission, and one had died from urosepsis. Disease responses were determined based on improvements in radiologic findings on FDG-PET imaging, characterized by reductions in metabolic activity and/or lesion size across previously involved sites. An overview of the treatment courses is provided (**Figure 3**).

Among the six patients undergoing treatment with confirmed BRAF V600E mutations, four received vemurafenib as a first-line therapy, and two were switched to vemurafenib after initially receiving other agents and later being found to be BRAF V600E-positive. At the time of follow-up, two of these patients had stable disease, two were in remission, and two had progressive or end-stage disease. Five patients reported side effects, including musculoskeletal pain (n=2), renal dysfunction (n=2), Dupuytren’s contracture (n=1), and photosensitivity (n=1), and two patients discontinued vemurafenib due to toxicity or disease progression.

Among the three patients who received pegylated interferon alpha-2a in their treatment course, stable disease was reported in two, and a partial response was reported in one patient. Treatment was limited by side effects, including anemia (n=1), vomiting (n=1), and infections (n=1), and a global drug shortage led to discontinuation in one case. Among the three patients who received tocilizumab during their treatment course, two had stable disease. One patient discontinued therapy after being found to be BRAF V600E positive, and one patient discontinued tocilizumab due to disease progression.

One patient received cobimetinib as a first-line therapy and remained on treatment with stable disease at last follow-up. Cladribine was used in two patients: one received it as a first-line therapy but was later found to be BRAF-positive and switched to targeted treatment; the other received cladribine as a second-line agent, with no clinical response and discontinued therapy due to poor performance status. One patient received cytarabine as a first-line therapy and had a partial response, though at the time of data cutoff, they had progressive disease.

Serial PET-CTs provided valuable information on treatment response and disease progression. Several patients demonstrated partial therapeutic responses or metabolic stability, including reduced FDG uptake in bone lesions (n=2), resolution of previously avid lymph nodes or soft tissue disease (n=2), and decreased pericardial or pleural involvement (n=2). PET-CT also helped identify possible disease progression in two patients, showing new hypermetabolic bone marrow foci and increased splenic activity.

## Discussion

ECD is a rare histiocytic neoplasm with highly heterogeneous clinical presentations leading to challenges and delays in diagnosis, which was seen in our single-center Canadian cohort, where patients experienced a median time to diagnosis of 12 months. This reflects the broader literature describing the highly variable clinical manifestations of ECD and the frequent need for multidisciplinary evaluation to reach a definitive diagnosis.^4,8,13,17,18^

Although bone involvement and bilateral long bone osteosclerosis are classically associated with ECD,^9,17–19^ our findings highlight the prominence of non-osseous manifestations such as retroperitoneal fibrosis, perirenal infiltration, cardiac involvement, and CNS disease. Notably, our results highlight the value of early FDG-PET/CT imaging in an ECD workup, and suggest that standardized imaging protocols may play a role in timely diagnosis and staging. However, access to PET-CT and other guideline-recommended imaging such as cardiac and brain MRI for histiocytosis have historically been limited in our centres. Our results highlight real-world limitations, where availability of resources may be more constrained, compared to larger centres specializing in histiocytosis care and research. One of the purposes of this study is also to improve adherence to guidelines for investigation and treatment of ECD.

Establishing a histopathologic diagnosis of ECD in this cohort was often difficult, due to the disease’s heterogeneous and frequently subtle presentation. Classic features were only seen in the first biopsy for four patients, and most required multiple biopsies (median: 2; range: 1-5). In the remaining seven cases, additional biopsies were pursued due to factors such as extensive fibrosis limiting histiocytic visibility.

Molecular profiling has fundamentally changed the understanding and management of ECD. Next-generation sequencing has become essential for identifying actionable mutations in the MAPK pathway, including BRAFV600E and MAP2K1, which directly inform the use of targeted therapies.^3,8,10^ Cohort analyses have also identified distinct clinical and mutational clusters, with BRAFV600E and multisystem disease being associated with poorer outcomes.^6,11^ In our cohort, molecular testing provided supportive but not definitive evidence in all cases. BRAF V600E mutations, reported in approximately 50-70% of ECD cases in the literature,^8,19,20^ were identified in seven patients (64%). In some cases, alternative somatic mutations were identified, including variants in TET2, U2AF1, and CSF3R, which align with the now well-established role of MAPK pathway dysregulation in ECD pathogenesis.^8,10,11^ Several patients had no identifiable pathogenic variants, reflecting the limitations of molecular panels. Establishing a molecular profile can be difficult in these cases, as bone is a common site of biopsy, and yet the decalcification process for bone specimens degrades DNA. Immunohistochemistry for BRAF, which detects BRAF activating mutations, can be helpful.

Treatment approaches varied widely based on BRAFV600E status, tolerability, comorbidities, and disease severity. Targeted therapy using BRAF inhibitors such as vemurafenib has demonstrated objective response rates of 55% in ECD, with a two-year progression-free survival of 86% and overall survival of 96%.^21^ For patients with BRAF-negative disease or other MAPK pathway mutations, MEK inhibitors such as cobimetinib and trametinib have shown comparable efficacy, achieving response rates up to 89% with durable disease control.^22,23^ Recent data further support that MEK inhibitor efficacy is significantly associated with the presence of MAPK pathway mutations, with lower response rates observed in mutation-negative patients.^24^ In our cohort, patients with BRAF activating mutations were treated with vemurafenib as the first line agent, leading to response in most. However, toxicities such as musculoskeletal pain, photosensitivity, Dupuytren’s and Peyronie’s contractures, can be difficult to manage and contributed to discontinuation in some cases. To address this, recent literature has proposed the use of “treatment holidays” from BRAF inhibitors, for example, one week on treatment followed by one week off, which helps mitigate adverse effects.^25,26^ Two patients in our cohort were on a one week on, one week off regimen. Interferon-alpha, historically a standard initial therapy for ECD,^8,27–29^ was used less frequently.

In some patients with ECD, severe systemic inflammation is the main clinical problem. For these patients, cytokine blockade with IL-6 inhibitors (tocilizumab, silutiximab) or an IL-1 receptor antagonist (anakinra) can be considered. Moreover, a recent study has shown that anakinra adjunctive therapy can enable patients to continue vemurafenib despite side effects.^30^ By targeting the inflammatory component of disease, anakinra may serve as a valuable adjunct to vemurafenib, helping to manage side effects while addressing the dual pathophysiology of ECD involving both myeloid cell infiltration of tissue and cytokine-mediated autoinflammation.

Strengths of this study include detailed patient-level data and a relatively long median follow-up time of four years. This allowed for the assessment of longitudinal treatment responses, disease progression, and tolerability of targeted therapies in a real-world setting. Limitations include the small sample size, retrospective design, and inherent heterogeneity of clinical and molecular features. Given the rarity of ECD, the lack of standardized treatment protocols and imaging intervals may have contributed to variation in reported outcomes and therapeutic responses. In addition, not all patients underwent comprehensive genomic testing, which may have led to underreporting of non-BRAF mutations with potential prognostic or therapeutic significance. Future directions may include the development of multicenter registries, improved access to genomic testing, and exploration of novel immunomodulatory therapies targeting IL-1, IL-6, or TNF-α pathways for treatment-resistant disease.

Overall, this single-center cohort study highlights the clinical heterogeneity and management challenges of ECD within a publicly funded healthcare system. Despite advances in molecular diagnostics and targeted therapies, ECD remains a disease with significant morbidity. Even within a small cohort, the diversity of presentations, diagnostic complexity, and treatment responses reflect the broader challenges of rare disease care, including limited access to FDG-PET imaging and unequal availability of novel therapies. The need for clearer diagnostic criteria and standardized treatment algorithms remains evident, particularly for patients without BRAF V600E mutations or those unable to tolerate vemurafenib.

### Authors’ Contribution

Conceptualization: LYCC; Methodology: LYCC; Data curation: LYCC, SQ, MT, LW; Formal analysis: LYCC, SQ; Writing - original draft: LYCC, SQ, EL, MG; Writing - review and editing: LYCC, FA, ML, MC; Supervision: LYCC

### Competing of Interest – COPE

Luke Y.C. Chen has received honoraria from Recordati Rare Diseases.

### Ethical Conduct Approval – Helsinki – IACUC

This retrospective study was approved by the University of British Columbia Clinical Research Ethics Board (H23-01853). All patient data was obtained from electronic medical records and anonymized. Given the retrospective nature of the study, the requirement for informed consent was waived by the ethics board.

### Informed Consent Statement

All authors have approved the final version of this manuscript.

## Data Availability

Data is available upon reasonable request to the corresponding author.
